# The −675 4G/5G Polymorphism in Plasminogen Activator Inhibitor-1 Gene Is Associated with Risk of Asthma: A Meta-Analysis

**DOI:** 10.1371/journal.pone.0034385

**Published:** 2012-03-27

**Authors:** Wei Nie, Bing Li, Qing-yu Xiu

**Affiliations:** Department of Respiratory Disease, Shanghai Changzheng Hospital, Second Military Medical University, Shanghai, China; Leibniz-Institute for Arteriosclerosis Research at the University Muenster, Germany

## Abstract

**Background:**

A number of studies assessed the association of −675 4G/5G polymorphism in the promoter region of *plasminogen activator inhibitor (PAI)-1* gene with asthma in different populations. However, most studies reported inconclusive results. A meta-analysis was conducted to investigate the association between polymorphism in the *PAI-1 g*ene and asthma susceptibility.

**Methods:**

Databases including Pubmed, EMBASE, HuGE Literature Finder, Wanfang Database, China National Knowledge Infrastructure (CNKI) and Weipu Database were searched to find relevant studies. Odds ratios (ORs) with 95% confidence intervals (CIs) were used to assess the strength of association in the dominant model, recessive model, codominant model, and additive model.

**Results:**

Eight studies involving 1817 cases and 2327 controls were included. Overall, significant association between 4G/5G polymorphism and asthma susceptibility was observed for 4G4G+4G5G vs. 5G5G (OR = 1.56, 95% CI 1.12–2.18, *P* = 0.008), 4G/4G vs. 4G/5G+5G/5G (OR = 1.38, 95% CI 1.06–1.80, *P* = 0.02), 4G/4G vs. 5G/5G (OR = 1.80, 95% CI 1.17–2.76, *P* = 0.007), 4G/5G vs. 5G/5G (OR = 1.40, 95% CI 1.07–1.84, *P* = 0.02), and 4G vs. 5G (OR = 1.35, 95% CI 1.08–1.68, *P* = 0.008).

**Conclusions:**

This meta-analysis suggested that the −675 4G/5G polymorphism of *PAI-1* gene was a risk factor of asthma.

## Introduction

Asthma is one of the most common chronic diseases affecting an estimated 300 million people worldwide [1]. Currently, it is recognized that asthma is a complex disease that results from interactions between multiple genetic and environmental factors [2]. Numerous studies have focused on the association between genetic variants and asthma risk, and the plasminogen activator inhibitor (PAI)-1 gene has been extensively studied.

PAI-1, a key regulator of the fibrinolytic system, is involved in various physiological functions and associated with many diseases. Kelly et al. [3] reported that PAI-1 mRNA expression was up-regulated in the bronchial wall at two weeks after chronic allergen exposure in the asthmatic mice. In house dust mite-sensitive allergic asthmatics, the average plasma level of PAI-1 was 49.7 ng/mL, and in healthy controls it was only 26.0 ng/mL (*P*<0.0001) [4]. Taken together, these facts suggested that PAI-1 may play an important role in pathogenesis of asthma.

The human *PAI-1* gene is located on chromosome 7 (q21.3–q22), where a locus related to serum IgE levels has been mapped [5]. The 4G/5G polymorphism (dbSNP rs1799889), which is characterized by a single guanosine nucleotide insertion/deletion variation at −675 bp of the *PAI-1* promoter, has been identified. The 4G/5G polymorphism is a major genetic determinant of plasma PAI-1 levels [6] and has been studied as a potential susceptibility factor for asthma. Several studies reported the association between the 4G/5G polymorphism of *PAI-1* gene and the risk of asthma [7]–[14], but the results were inconclusive.

Considering a single study may lack the power to provide reliable conclusion, we performed a meta-analysis to investigate the precise relationship between the *PAI-1* gene variants and asthma. This was, to our knowledge, the first meta-analysis of the association between *PAI-1* polymorphism and asthma susceptibility.

## Methods

### Publication search

Pubmed, EMBASE, HuGE Literature Finder, Wanfang Database, China National Knowledge Infrastructure (CNKI) and Weipu Database were all searched (Last search was updated on October, 2011). The following terms were used in searching: (asthma or asthmatic) and (plasminogen activator inhibitor-1 or PAI-1 or SERPINE1) and (polymorphism or mutation or variant). No publication date or language restrictions were imposed. All the searched studies were retrieved, and their references were checked as well for other relevant publications. Review articles were also searched to find additional eligible studies.

### Inclusion and Exclusion Criteria

Studies fulfilling the following selection criteria were included in this meta-analysis: (1) evaluation of the 4G/5G polymorphism in *PAI-1* gene and asthma risk, (2) using a case-control design, (3) genotype distributions in both cases and controls should be available for estimating an odds ratio (OR) with 95% confidence interval (CI), and (4) genotype distribution of control group must be consistent with Hardy–Weinberg equilibrium (HWE). Studies were excluded if one of the following existed: (1) not relevant to PAI-1 or asthma, (2) the design based on family or sibling pairs, (3) genotype frequencies or number not reported, and (4) reviews and abstracts. For overlapping studies, only the one with the largest sample numbers was included.

### Data Extraction

Two investigators (Nie and Li) independently reviewed full manuscripts of eligible studies, and the relevant data were extracted into predesigned data collection forms. We verified accuracy of data by comparing collection forms from each investigator. Any discrepancy was resolved by discussion or a third author (Xiu) would assess these articles. The following variables were collected from each study: first author's name, year of publication, original country, ethnicity, sample size, asthma definition, genotyping method, atopic status, and genotype numbers in cases and controls.

### Statistical Analysis

The strength of the association between the 4G/5G polymorphism and asthma risk was measured by OR and 95% CI. The statistical significance of OR was analyzed by *Z* test, and *P*<0.05 was considered as statistically significant. We first estimated with the dominant model (4G4G+4G5G vs. 5G5G) and recessive model (4G4G vs. 4G5G+5G5G) and then evaluated codominant model (4G4G vs. 5G5G and 4G5G vs. 5G5G). We also estimated the risks of additive model (4G vs. 5G).

The heterogeneity between the studies was assessed by the Chi square-test based Cochrane *Q*-test. *I* ^2^ was also used to test the heterogeneity among the included studies. A *P* value>0.10 for the *Q*-test indicates a lack of heterogeneity among the studies, then the pooled OR estimate of each study was calculated by the fixed-effects model (the Mantel-Haenszel method). Otherwise, the random-effects model (the DerSimonian and Laird method) was used. In addition, a Chi square-test was used to determine if observed frequency of genotype in control population conformed to HWE expectations.

To evaluate the ethnic-specific, age-specific, and atopic-specific effects and to explore sources of heterogeneity, subgroup analyses were performed by ethnicity, age, and atopic status. Sensitivity analysis was performed through sequentially excluded individual studies to assess the stability of the results. Asymmetry funnel plots were used to assess potential publication bias. The Begg's test [15] and Egger's test [16] were also used to assess publication bias statistically.

All statistical tests were performed by using the Revman 5.1 software (Nordic Cochrane Center, Copenhagen, Denmark) and STATA 11.0 software (Stata Corporation, College Station, TX).

## Results

### Study Characteristics

**Figure 1:**
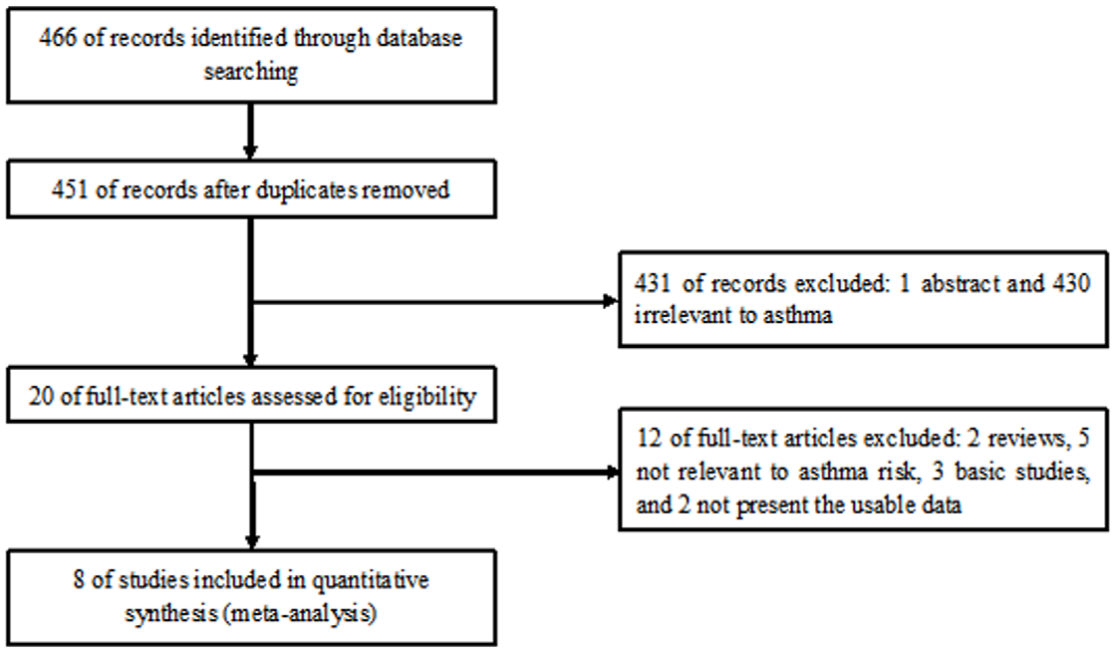
Flow of study identification, inclusion, and exclusion.

**Figure 1** outlines our study selection process. Briefly, a total of 466 articles were identified after an initial search. After removing duplications and reading the abstracts, 430 articles were excluded for not being relevant to asthma. One abstract was excluded because we could not get the full text. After reading full texts of the remaining 20 articles, twelve were then excluded and eight remained. One article reported two cohorts [14], and each cohort was considered as a separate case-control study. Finally, a total of 9 case-control studies in 8 articles were identified met our inclusion criteria [7]–[14], including 1817 cases and 2327 controls. There were 4 studies of Asians [9], [11]–[13] and 4 studies of Caucasians [7], [8], [10], [14]. Four studies were performed in adults [10], [12]–[14], one in children [11], and three included both children and adults [7]–[9]. Three studies only included atopic asthma patients [7], [8], [10], two studies included both of these patients [9], [12], and three studies did not offer detailed information [11], [13], [14]. The characteristics of each study included in this meta-analysis are presented in **Table S1**. Genotype numbers and HWE examination results are listed in **Table S2**.

### Quantitative Data Synthesis

#### All studies

**Figure 2:**
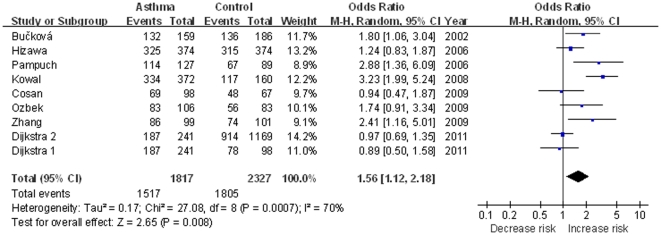
Meta-analysis with a random-effects model for the association between asthma risk and the *PAI-1* 4G/5G polymorphism (4G4G+4G5G vs. 5G5G).

As shown in **Figure 2**, we analyzed the heterogeneity of 4G4G+4G5G vs. 5G5G for all the 9 studies, and the value of χ^2^ was 27.08 and *P* = 0.0007 in a random effects model. In addition, the *I* ^2^ value was 70%, suggesting significant heterogeneity. Therefore, the random-effects model was used for synthesis of the data. The overall OR for 4G4G+4G5G genotypes versus 5G5G genotypes (dominant model) was 1.56 (95% CI 1.12–2.18) and the *Z* test for overall effect was 2.65 (*P* = 0.008). This result suggested that individuals who carry the 4G allele may have a 56% increased asthma risk compared with 5G5G homozygote. When all the studies were pooled into meta-analysis using other genetic models (**Table S3**), there was also significant association between 4G/5G and asthma risk (for recessive model: OR = 1.38, 95% CI 1.06–1.80, *P* = 0.02; for codominant model: OR = 1.80, 95% CI 1.17–2.76, *P* = 0.007, and OR = 1.40, 95% CI 1.07–1.84, *P* = 0.02; for addictive model: OR = 1.35, 95% CI 1.08–1.68, *P* = 0.008).

#### Subgroup analyses

In the subgroup analyses by ethnicity, significant associations were found among Asians (OR 1.40, 95% CI 1.06–1.86, *P* = 0.02) but not among Caucasians (OR 1.66, 95% CI 0.97–2.86, *P* = 0.07) in dominant model (4G4G+4G5G vs. 5G5G). Thus, Asian carriers of the 4G allele may have an increased risk of asthma. In the subgroup analyses by age, significant associations were found among adults (OR 1.61, 95% CI 1.04–2.50, *P* = 0.03), suggesting that adults carriers of 4G allele may have a 61% increased risk of asthma. Subgroup analyses were also performed by atopic status. Significant increased risk of asthma was found among atopic asthma patients (OR 2.51, 95% CI 1.81–3.46, *P*<0.00001). The result suggested that the 4G allele carriers (4G4G+4G5G) have an increased risk of atopic asthma compared with those individuals with the 5G5G homozygote. Furthermore, the heterogeneity was decreased significantly in atopic subgroup in every genetic model, suggesting atopic status was the potentially important source of heterogeneity. Summary results of other genetic comparisons are listed in **Table S3**.

#### Sensitivity Analysis

In order to assess the stability of the results of the meta-analysis, we performed a sensitivity analysis through sequentially excluded individual studies. Statistically similar results were obtained after sequentially excluding each study, suggesting stability of the meta-analyses.

#### Publication bias

**Figure 3:**
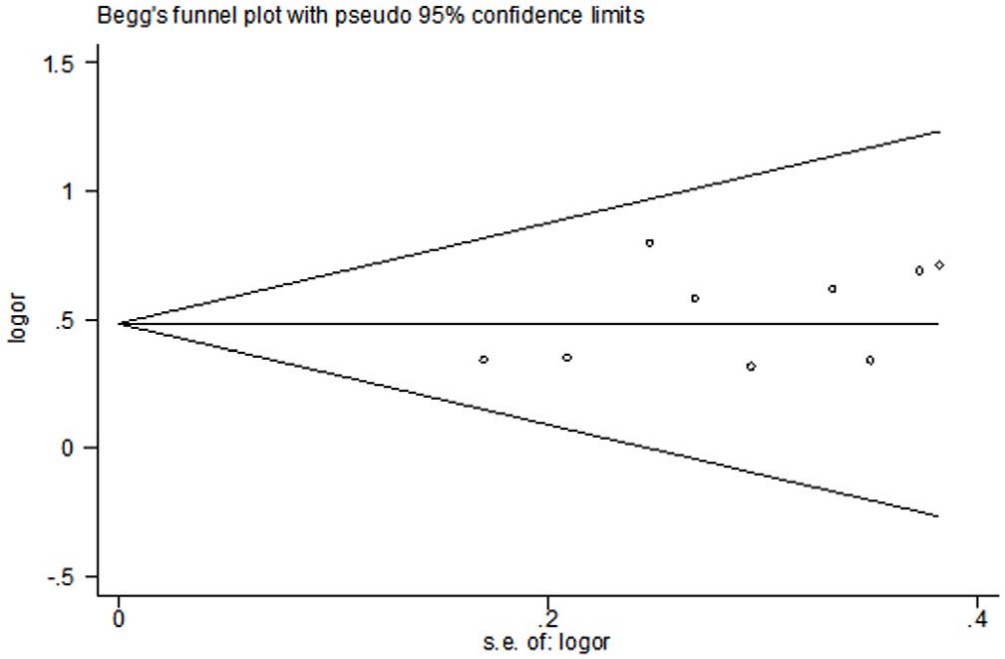
Begg's funnel plot for publication bias in selection of studies on the *PAI-1* 4G/5G polymorphism (4G4G+4G5G vs. 5G5G).

Publication bias was assessed by Begg's funnel plot and Egger's test. The shape of the funnel plot showed slightly asymmetric in the 4G4G+4G5G vs. 5G5G comparison genetic model, suggesting the possibility of publication bias (**Figure 3**). Then, the Egger's test was performed to provide statistical evidence of funnel plot asymmetry. The results indicated a lack of publication bias of the current meta-analysis (*t* = 1.48, *P* = 0.182).

## Discussion

Asthma is a genetically complex disease caused by multiple genetic and environmental factors and is the result of the interaction of multiple genetic and environmental factors [17]. The pathophysiologic hallmark of asthma is chronic inflammation leading to airway hyper-responsiveness (AHR), mucous hypersecretion and remodeling of airways [18]. Recent studies suggested that PAI-1 may promote the development of asthma by facilitating AHR, allergic inflammation, and airway remodeling [19]. For instance, Savov et al. [20] reported that four weeks after lipopolysaccharide inhalation, AHR and the expansion of the subepithelial area in the medium and large airways were observed in WT but not in *PAI-1* deficient mice. In a murine allergic rhinitis model, the immune response appeared to be a dominant Th2 response in WT mice; however, in *PAI-1* deficient mice, the Th2 response was shifted to a Th1-dominant response, suggesting a key role of PAI-1 in allergic inflammation [21]. In addition, PAI-1 promoted extracellular matrix (ECM) deposition in the asthmatic airways. After OVA challenge, the levels of collagen deposition were approximately 50% less in lung tissues from *PAI-1* deficient mice compared with WT mice [22]. These evidences supported that PAI-1 may play an essential role in the pathogenesis of asthma. Among SNPs of the *PAI-1* gene, the 4G/5G polymorphism has been extensively studied. The 4G/4G genotype has been linked to higher PAI-1 level, compared with the 5G/5G genotype, with the heterozygous genotype associated with intermediate levels [23]. It is biologically plausible that 4G/5G polymorphism which affects PAI-1 level could influence the susceptibility to asthma.

This current meta-analysis of 8 studies including 1817 cases and 2327 controls systematically evaluated the association between −675 4G/5G polymorphism in the *PAI-1* gene promoter and asthma risk. The results indicated that −675 4G/5G polymorphism was a conspicuous high risk factor for developing asthma in the overall study populations. In the subgroup analysis by ethnicity, no significant association was found in Caucasians in dominant model. However, asthma risk was increased in Asians (*P* = 0.02), suggesting a possible influence among environmental exposures and different genetic backgrounds. Cho et al. [24] reported that the 4G allele of the *PAI-1* gene may be associated with the development of asthma in children. However, significant associations were only found in adults (*P* = 0.03). Since this meta-analysis included only one study using children population, the positive association between children and asthma could not be ruled out because studies with small sample size may have insufficient statistical power to detect a slight effect. Additional future studies should be performed focusing on children with asthma. Subgroup analysis was also performed among atopic status patients. Significant increased risk of asthma was found in these patients (*P*<0.00001), suggesting a possible role of PAI-1 in the etiology of allergic asthma. In addition, all atopic status patients were Caucasians. More studies are warranted to further validate atopic status difference in the effect of this polymorphism on asthma susceptibility, especially in Asians.

As the publication of findings often depends on the expectation of researchers, false-negative results may be suppressed or false-positive results magnified [25]. The results of this study, however, did not show significant publication bias. However, since the number of studies included in this meta-analysis was small and large interstudy heterogeneity was observed. Significant heterogeneity existed in overall comparisons in each genetic model. The observed heterogeneity could be attributable to differences in several factors such as atopic status, ethnic variations, environmental factors and methodological factors in design and conduct of the studies. Among these factors, atopic status could play a crucial role. After subgroup analysis by atopic status, the heterogeneity was effectively decreased in atopic asthma patients. Therefore, it can be presumed that the relatively large heterogeneity mainly results from differences of atopic status.

The present meta-analysis had several limitations that must be taken into account. First, the number of available studies that could be included in this meta-analysis was moderate. Therefore, the results could be influenced by the factors like random error. Second, the overall outcomes were based on individual unadjusted ORs, while a more precise evaluation should be adjusted by other potentially suspected factors including age, sex, and environmental factors. Third, because of the complex nature of asthma, it is unlikely that a SNP in one single gene would be obviously associated with an increase in asthma risk, without consideration of any other polymorphic susceptible genes. Kowal et al. [10] pointed out that the synergistic interactions of the 4G/5G polymorphism with the CD14 C-159T polymorphism affected susceptibility and severity of house dust mite-allergic asthma. Thus, we could not rule out the possibility of SNP–SNP interactions. Fourth, there was no study of American African population and only one study of children in this meta-analysis. Finally, since the number of studies included in the subgroup analyses was small, the results lacked sufficient reliability to confirm or refute an association in a definitive manner.

To our knowledge, this study was the first comprehensive meta-analysis to assess the relationship between the *PAI-1* 4G/5G polymorphism and asthma susceptibility. It provided evidence of the association between *PAI-1* −675 4G/5G polymorphism and asthma risk, supporting the hypothesis that the *PAI-1* −675 4G/5G polymorphism may be a susceptibility marker for asthma. However, additional large case-control studies are required to validate our findings. Future analyses should be conducted in large-scale cohorts and should study the potential effect modification by age and atopic status in different populations. Moreover, gene-gene and gene-environment interactions should also be considered in future studies.

## Supporting Information

Table S1 **Characteristics of the 9 case-control studies included in meta-analysis.**
(DOC)

Table S2 **Distribution of** ***PAI-1*** **genotype among patients with asthma and controls included in the meta-analysis.**
(DOC)

Table S3 **Summary of different comparative results.**
(DOC)
