# Supplementary material for: The −675 4G/5G Polymorphism in Plasminogen Activator Inhibitor-1 Gene Is Associated with Risk of Asthma: A Meta-Analysis
Source: PLoS One. 2012 Mar 27;7(3):e34385. doi: 10.1371/journal.pone.0034385 (PMC3313978; doi:10.1371/journal.pone.0034385)
Supplement: Table S1 — Characteristics of the 9 case-control studies included in meta-analysis. (DOC) [file pone.0034385.s001.doc]

**Table S1**. Characteristics of the 9 case-control studies included in meta-analysis.*

| First author | Year | Country | Ethnicity | Adults / | Case | Control | Asthma definition | Atopic | Genotyping |
| --- | --- | --- | --- | --- | --- | --- | --- | --- | --- |
|  |  |  |  | Children | number (n) | number (n) |  | status | method |
| Bučková [7] | 2002 | Czech | Caucasian | Adults | 159 | 186 | Questionnaire and physician’s diagnosis | Atopic | PCR |
| Pampuch [8] | 2006 | Poland | Caucasian | Adults | 127 | 89 | GINA criteria | Atopic | PCR |
| Hizawa [9] | 2006 | Japan | Asian | Mixed | 374 | 374 | Clinical diagnosis | Mixed | PCR |
| Kowal [10] | 2008 | Poland | Caucasian | Adults | 372 | 160 | GINA criteria | Atopic | PCR |
| Ozbek [11] | 2009 | Turkey | Asian | Children | 106 | 83 | ATS criteria | NA | PCR |
| Cosan [12] | 2009 | Turkey | Asian | Adults | 98 | 67 | ATS criteria | Mixed | PCR |
| Zhang [13] | 2009 | China | Asian | Adults | 99 | 101 | Chinese asthma diagnosis criteria | NA | PCR |
| Dijkstra 1 [14] | 2011 | Netherlands | Caucasian | Adults | 241 | 98 | Asthma algorithm | NA | PCR |
| Dijkstra 2 [14] | 2011 | Netherlands | Caucasian | Adults | 241 | 1169 | Asthma algorithm and medical history | NA | PCR |

* PCR, polymerase chain reaction; ATS, American Thoracic Society; GINA, Global Initiative for Asthma; NA, not available.
