# Supplementary material for: The −675 4G/5G Polymorphism in Plasminogen Activator Inhibitor-1 Gene Is Associated with Risk of Asthma: A Meta-Analysis
Source: PLoS One. 2012 Mar 27;7(3):e34385. doi: 10.1371/journal.pone.0034385 (PMC3313978; doi:10.1371/journal.pone.0034385)
Supplement: Table S2 — Distribution of PAI-1 genotype among patients with asthma and controls included in the meta-analysis. (DOC) [file pone.0034385.s002.doc]

**Table S2**. Distribution of *PAI-1* genotype among patients with asthma and controls included in the meta-analysis.

| Studies | Asthma | | |  | Control | | | Hardy–Weinberg |
| --- | --- | --- | --- | --- | --- | --- | --- | --- |
|  | 4G/4G | 4G/5G | 5G/5G |  | 4G/4G | 4G/5G | 5G/5G | equilibrium |
| Bučková [7] | 57 | 75 | 27 |  | 53 | 83 | 50 | Yes |
| Pampuch [8] | 61 | 53 | 13 |  | 31 | 36 | 22 | Yes |
| Hizawa [9] | 131 | 194 | 49 |  | 130 | 185 | 59 | Yes |
| Kowal [10] | 180 | 154 | 38 |  | 47 | 70 | 43 | Yes |
| Ozbek [11] | 44 | 39 | 23 |  | 15 | 41 | 27 | Yes |
| Cosan [12] | 26 | 43 | 29 |  | 19 | 29 | 19 | Yes |
| Zhang [13] | 37 | 49 | 13 |  | 28 | 46 | 27 | Yes |
| Dijkstra 1 [14] | 70 | 117 | 54 |  | 31 | 47 | 20 | Yes |
| Dijkstra 2 [14] | 70 | 117 | 54 |  | 334 | 580 | 255 | Yes |
