# Supplementary material for: The −675 4G/5G Polymorphism in Plasminogen Activator Inhibitor-1 Gene Is Associated with Risk of Asthma: A Meta-Analysis
Source: PLoS One. 2012 Mar 27;7(3):e34385. doi: 10.1371/journal.pone.0034385 (PMC3313978; doi:10.1371/journal.pone.0034385)
Supplement: Table S3 — Summary of different comparative results. (DOC) [file pone.0034385.s003.doc]

**Table S3**. Summary of different comparative results.*

|  | Study | Sample size | | No. of | Test of association | | | Model | | Heterogeneity | | |
| --- | --- | --- | --- | --- | --- | --- | --- | --- | --- | --- | --- | --- |
|  |  | case | control | studies | OR (95% CI) | *Z* | *P* Value | |  | *χ*^2^ | *P* Value | *I*^2^ (%) |
| 4G/4G + 4G/5G vs. 5G/5G | Overall | 1817 | 2327 | 9 | 1.56 (1.12 – 2.18) | 2.65 | 0.008 | | R | 27.08 | 0.0007 | 70.0 |
|  | Asian | 677 | 625 | 4 | 1.40 (1.06 – 1.86) | 2.34 | 0.02 | | F | 4.17 | 0.24 | 28.0 |
|  | Caucasian | 1140 | 1702 | 5 | 1.66 (0.97 – 2.86) | 1.84 | 0.07 | | R | 22.84 | 0.0001 | 82.0 |
|  | Adults | 1337 | 1870 | 7 | 1.61 (1.04 – 2.50) | 2.16 | 0.03 | | R | 26.22 | 0.0002 | 77.0 |
|  | Atopic | 658 | 435 | 3 | 2.51 (1.81 – 3.46) | 5.57 | < 0.00001 | | F | 2.72 | 0.26 | 27.0 |
| 4G/4G vs. 4G/5G + 5G/5G | Overall | 1817 | 2327 | 9 | 1.38 (1.06 – 1.80) | 2.36 | 0.02 | | R | 23.92 | 0.002 | 67.0 |
|  | Asian | 677 | 625 | 4 | 1.42 (0.85 – 2.37) | 1.33 | 0.18 | | R | 10.62 | 0.01 | 72.0 |
|  | Caucasian | 1140 | 1702 | 5 | 1.37 (0.97 – 1.94) | 1.78 | 0.08 | | R | 12.00 | 0.01 | 69.0 |
|  | Adults | 1337 | 1870 | 7 | 1.34 (1.01 – 1.77) | 2.03 | 0.04 | | R | 14.39 | 0.03 | 58.0 |
|  | Atopic | 658 | 435 | 3 | 1.82 (1.40 – 2.36) | 4.48 | < 0.00001 | | F | 2.42 | 0.30 | 17.0 |
| 4G/4G vs. 5G/5G | Overall | 976 | 1210 | 9 | 1.80 (1.17 – 2.76) | 2.70 | 0.007 | | R | 34.55 | < 0.0001 | 77.0 |
|  | Asian | 352 | 324 | 4 | 1.73 (0.96 – 3.13) | 1.81 | 0.07 | | R | 8.42 | 0.04 | 64.0 |
|  | Caucasian | 624 | 886 | 5 | 1.85 (0.96 – 3.58) | 1.84 | 0.07 | | R | 26.07 | < 0.0001 | 85.0 |
|  | Adults | 729 | 979 | 7 | 1.77 (1.05 – 3.01) | 2.13 | 0.03 | | R | 29.61 | < 0.0001 | 80.0 |
|  | Atopic | 376 | 246 | 3 | 3.06 (2.14 – 4.39) | 6.10 | < 0.00001 | | F | 3.60 | 0.17 | 44.0 |
| 4G/5G vs. 5G/5G | Overall | 1141 | 1639 | 9 | 1.40 (1.07 – 1.84) | 2.42 | 0.02 | | R | 15.96 | 0.04 | 50.0 |
|  | Asian | 439 | 433 | 4 | 1.29 (0.96 – 1.75) | 1.67 | 0.10 | | F | 2.59 | 0.46 | 0.0 |
|  | Caucasian | 702 | 1206 | 5 | 1.50 (0.96 – 2.34) | 1.79 | 0.07 | | R | 13.30 | 0.01 | 70.0 |
|  | Adults | 836 | 1327 | 7 | 1.48 (1.03 – 2.13) | 2.14 | 0.03 | | R | 15.59 | 0.02 | 62.0 |
|  | Atopic | 360 | 304 | 3 | 2.14 (1.51 – 3.02) | 4.33 | < 0.00001 | | F | 1.20 | 0.55 | 0.0 |
| 4G vs. 5G | Overall | 3634 | 4654 | 9 | 1.35 (1.08 – 1.68) | 2.64 | 0.008 | | R | 37.87 | < 0.00001 | 79.0 |
|  | Asian | 1354 | 1250 | 4 | 1.32 (0.95 – 1.83) | 1.66 | 0.10 | | R | 10.40 | 0.02 | 71.0 |
|  | Caucasian | 2280 | 3404 | 5 | 1.37 (0.98 – 1.91) | 1.84 | 0.07 | | R | 27.15 | < 0.0001 | 85.0 |
|  | Adults | 2674 | 3740 | 7 | 1.33 (1.02 – 1.74) | 2.11 | 0.04 | | R | 30.31 | < 0.0001 | 80.0 |
|  | Atopic | 1316 | 870 | 3 | 1.78 (1.49 – 2.13) | 6.31 | < 0.00001 | | F | 3.85 | 0.15 | 48.0 |

* vs.: versus; R: random-effects model; F: fixed-effects model.
